# Anti-Dengue IgG Seroprevalence and Exposure-Related Risk in Italian Military Personnel Deployed on Overseas Missions: A Cross-Sectional Study

**DOI:** 10.3390/tropicalmed11060167

**Published:** 2026-06-18

**Authors:** Andrea Ciammaruconi, Anna Rocchetti, Filippo Molinari, Elisa Recchia, Nathalie Totaro, Chiara Pascolini, Silvia Chimienti, Giovanni Faggioni, Riccardo De Santis, Filippo Moramarco, Alberto Autore, Florigio Lista

**Affiliations:** 1 Defence Institute for Biomedical Sciences, 00184 Rome, Italy; anna.rocchetti@persociv.difesa.it (A.R.); molinarifilippo@hotmail.com (F.M.); elisa.recchia@persociv.difesa.it (E.R.); nathalie.totaro@persociv.difesa.it (N.T.); chiara.pascolini@persociv.difesa.it (C.P.); silvia.chimienti@persociv.difesa.it (S.C.); giovanni.faggioni@gmail.com (G.F.); r.desantis@uniroma1.it (R.D.S.); filippo.moramarco@persociv.difesa.it (F.M.); florigio.lista@esercito.difesa.it (F.L.); 2 Dipartimento di Scienze e Tecnologie Chimiche, Università di Roma Tor Vergata, 00133 Rome, Italy; 3 Dipartimento di Chimica, Biologia e Biotecnologie, Università degli Studi di Perugia, 06123 Perugia, Italy; 4 Dipartimento di Scienze Cliniche e Medicina Traslazionale, Università di Roma Tor Vergata, 00133 Rome, Italy; 5 Department of Public Health and Infectious Diseases, Sapienza University of Rome, 00185 Rome, Italy; 6 Institute of Aerospace Medicine, Italian Air Force, 00185 Rome, Italy; alberto.autore@aeronautica.difesa.it

**Keywords:** dengue virus, dengue seroprevalence, military personnel, surveillance, machine learning, cross-sectional study

## Abstract

Dengue virus infection remains a significant public health challenge in endemic regions, with growing evidence of autochthonous transmission in Europe. Assessing serological exposure in high-risk populations such as military personnel deployed to endemic areas is essential to quantify exposure risk and support operational decision-making, particularly regarding pre-deployment counselling and risks associated with secondary infection. We conducted a cross-sectional study involving 1355 members of the Italian Armed Forces, measuring anti-dengue IgG antibodies by ELISA and collecting data on deployment history and exposure risk. Overall, IgG seropositivity was 8.12%, with significantly higher prevalence among individuals reporting travel or deployment to endemic regions (24.71%) compared with non-exposed personnel (4.27%). Seropositivity increased with age and correlated with a CDC-derived cumulative dengue risk score (Spearman’s ρ = 0.299, *p* < 0.0001). A multivariable logistic regression model including age and exposure risk achieved an AUC of 0.75, while machine-learning models provided complementary predictive assessment, with random forest reaching an AUC of 0.79. These findings indicate substantial anti-dengue IgG seropositivity compatible with previous dengue exposure among Italian military personnel, particularly those deployed to endemic settings. The study highlights the need for targeted surveillance and risk-based preventive strategies, and supports the use of exposure-based models to improve epidemiological assessment and inform vaccination strategies in mobile populations.

## 1. Introduction

Dengue is a mosquito-borne viral disease caused by dengue virus (DENV), a member of the *Flaviviridae* family within the genus Flavivirus. Transmission occurs through the bite of infected female Aedes mosquitoes, mainly *Aedes aegypti* and *A. albopictus*. The pathogen circulates widely across tropical and subtropical regions, with established endemicity in Southeast Asia, the Pacific Islands, the Americas, and large parts of Africa [1].

Mainland Europe remains non-endemic; nonetheless, imported infections are regularly documented. Italy recorded 1043 dengue cases between 2010 and 2023, most of them acquired during travel [2], while only 11 were classified as autochthonous during that period [3]. Local transmission has become more visible in several European countries over the past decade, including France, where autochthonous infections were reported in 2022, 2023, and 2024 [4,5]. Italy experienced a series of locally acquired cases as well. In 2020, transmission clusters were identified in the Veneto region [6,7], with subsequent reports from Lombardy [8] and Lazio [9]. The situation evolved further in 2023, when Italy registered 82 confirmed autochthonous cases [10], including multiple transmission episodes in the Lazio region [11,12,13], alongside 295 imported infections [14]. During the 2024 transmission season, 741 dengue cases were notified between late July and November; of these, 238 were autochthonous and distributed across six regions [15]. A substantial outbreak involving more than 199 dengue cases occurred in the town of Fano [16]. Data for 2025 point to persisting local transmission risks [17]. These data underscore the growing vulnerability of Europe to localised dengue outbreaks, a trend also driven by the expanding distribution of competent Aedes vectors.

Dengue virus comprises four antigenically distinct serotypes, designated DENV-1, DENV-2, DENV-3 and DENV-4 [18]. A primary infection induces serotype-specific antibodies that provide lasting protection only against the homologous serotype. When exposure to a different serotype occurs, pre-existing antibodies may enhance viral entry into Fc-receptor-bearing cells, a phenomenon known as antibody-dependent enhancement (ADE) [19], which has been associated with increased severity during secondary infection, including severe clinical manifestations such as shock, haemorrhage or organ dysfunction [20]. In this context, identifying previously exposed individuals in high-risk populations is essential for assessing the risk of severe disease, guiding preventive strategies, and supporting epidemiological surveillance.

Military personnel often face a higher probability of encountering multiple dengue serotypes because of frequent deployments to endemic regions and repeated travel through diverse ecological settings. A cross-sectional investigation conducted in 2003 by Peragallo et al. [21] illustrates this vulnerability: among Italian Army troops deployed to East Timor, 6.6% were found to have contracted probable dengue fever. Further evidence comes from the study by Hesse et al. [22], who analysed serum samples from U.S. service members returning from dengue-endemic areas and detected anti-dengue neutralising antibodies in 7.6% of individuals. A recent systematic review of dengue in deployed military personnel (1905–2024) selected 32 studies for final analysis, summarising more than a century of data on incidence, outbreak dynamics, diagnostics, and prevention strategies across multiple armed forces [23]. Notably, the majority of available evidence derives from deployments involving the United States and French armed forces. By contrast, data from Italian military personnel remain extremely limited, with the investigation conducted in East Timor representing the only report of dengue exposure in this population. This imbalance underscores the scarcity of contemporary seroepidemiological data from European military cohorts, highlighting the need to reassess dengue exposure patterns in contemporary military settings.

The present study evaluates the seroprevalence of anti-dengue IgG in a defined subset of the Italian Armed Forces undergoing pre-deployment assessment for overseas missions. The primary objective was to estimate anti-dengue IgG seroprevalence in this population. Secondary objectives were to assess the association between IgG seropositivity and previous travel or deployment to dengue-endemic regions, to identify geographical areas associated with higher exposure-related risk, and to evaluate the relationship between seropositivity and a CDC-derived cumulative dengue risk score. This information may support exposure-based risk stratification and help inform targeted preventive strategies in mobile military populations.

While earlier machine-learning work on dengue has largely examined environmental or climatic drivers of transmission [24,25], this study applies supervised machine-learning techniques to individual-level demographic and exposure data. By combining traditional statistical models with machine-learning frameworks, the study aims to better delineate the determinants of IgG seropositivity, identify operational risk profiles and reinforce health-surveillance systems for deployed personnel.

## 2. Materials and Methods

### 2.1. Study Design and Population

This cross-sectional study took place between December 2022 and August 2023 and involved Italian military personnel stationed in different regions of the country prior to deployment to dengue-endemic areas. Eligible subjects were active-duty personnel aged 18 years or older.

Participants were enrolled during routine pre-deployment occupational health and surveillance activities conducted by military medical personnel. Written informed consent was obtained before questionnaire administration and blood sampling. Each participant completed a physician-assisted structured questionnaire in paper format, administered prior to serological testing. The questionnaire collected demographic information and data on previous travel or deployment to dengue-endemic countries or regions. The collected information was used for exposure classification and cumulative risk-score calculation.

### 2.2. Ethics Statement

The study was conducted in accordance with the principles of the Declaration of Helsinki and relevant national regulations. All participants provided written informed consent both for the performance of clinical laboratory investigations within routine occupational health and surveillance activities of the Italian Armed Forces and for the secondary use of their anonymised personal and clinical data for research purposes.

Analyses were performed exclusively on anonymised datasets. Personal data were processed in compliance with Italian Legislative Decree No. 196/2003 and the European Union General Data Protection Regulation (GDPR, Regulation EU 2016/679). Ethical review and approval were waived for this study, because the research involved the secondary analysis of fully anonymised data collected within routine occupational health and surveillance activities, in accordance with applicable national regulations.

### 2.3. Serological Testing

Peripheral blood samples were collected during the pre-deployment health assessment at the military medical facilities where personnel were evaluated. Venous blood was collected in serum separator tubes with a separation gel and centrifuged to obtain serum. Serum samples were stored at −20 °C before analysis and transported to the Defence Institute for Biomedical Sciences, Rome, Italy, according to standard procedures and applicable Italian and international regulations for the transport of biological specimens. All serological analyses were performed at the Defence Institute for Biomedical Sciences, a research centre of the Italian Ministry of Defence.

Serum samples were tested for IgG antibodies against DENV serotypes 1–4 using a commercial enzyme-linked immunosorbent assay (Dengue Virus IgG ELISA TECAN, IBL International GmbH, Hamburg, Germany). The assay is semi-quantitative and includes positive, negative and cut-off controls run in duplicate. Samples were diluted 1:100 and results were expressed in NovaTec Units (NTU), calculated as the mean absorbance multiplied by 10. According to the manufacturer’s instructions, results were classified as negative (<9 NTU), equivocal (9–11 NTU), or positive (>11 NTU). Equivocal samples were retested, and if the result remained equivocal, classified as negative. The assay has a diagnostic specificity of 95.87% (95% CI: 90.62–98.64%) and a sensitivity of 100% (95% CI: 93.62–100%), as specified by the manufacturer.

Anti-dengue IgG serostatus was defined according to the manufacturer’s cut-off values and used as the serological outcome for subsequent analyses.

### 2.4. Statistical Analysis

Data analysis was conducted with IBM SPSS Statistics for Windows, version 26.0 (IBM Corp., United States). The dataset used for all statistical analyses is provided as Supplementary S1_Data, which includes anonymized individual-level variables such as age, sex, Armed Forces branch, deployment history, cumulative exposure risk score, and anti-dengue IgG serostatus. Seroprevalence of anti-dengue IgG antibodies was calculated for the entire sample and across exposure groups. Associations between seropositivity and categorical variables were assessed using Pearson’s chi-square test with Yates’ continuity correction when appropriate. Odds ratios (ORs) with 95% confidence intervals (CIs) were computed from 2 × 2 contingency tables to quantify effect size. Two-tailed *p*-values ≤ 0.05 were considered statistically significant.

### 2.5. Exposure Risk Scores Definition

Risk scores for each deployment were categorised based on the Centers for Disease Control and Prevention (CDC) dengue risk classification [26]. We developed a semi-quantitative scoring system by assigning ordinal weights to different risk levels (Country_Risk_Classification in S1_Data). Regions with “Variable regional risk” were assigned a score of 1, those with “Sporadic or uncertain” risk a score of 2, and areas with “Frequent and continuous” risk a score of 3. These values were then squared, obtaining final weights of 1, 4, and 9, thus enhancing separation between exposure categories and better reflecting the non-linear increase in transmission risk. In cases where travel history was limited to broad geographical regions (e.g., Western, Central, or Eastern Africa), an intermediate baseline score of 2.5 was assigned, in order to reflect uncertainty between moderate and high transmission risk categories. This value was then squared, yielding a final weight of 6.25. The cumulative exposure risk for each participant was calculated as the sum of the weights corresponding to all visited locations. This indexing approach serves as a pragmatic proxy for cumulative exposure, enabling relative epidemiological comparisons between individuals rather than providing a precise estimation of absolute infection risk.

### 2.6. Machine-Learning Analysis

Supervised machine-learning models were developed to examine the predictive value of demographic and exposure-related variables for IgG seropositivity. After cleaning and preprocessing, the dataset was randomly split into training (80%) and test (20%) subsets using stratified sampling to preserve class proportions. Logistic regression, random forest, naïve Bayes and support vector machine (SVM) classifiers were trained and evaluated through five-fold cross-validation with balanced class weights to address class imbalance. Hyperparameters were tuned via randomised grid search. Model performance was assessed using the area under the receiver operating characteristic (ROC) curve (AUC) and the F1-score.

Exploratory principal component analysis (PCA) was performed on four standardised variables (Age, Risk_Level, Sex and Armed_Forces) to examine underlying structural patterns in the data. Reproducible code for all analytical steps is provided in S2_File.

For this study, the integrated development environment (IDE) employed was Google Colab, configured with a Python 3 runtime environment and hosted on a Google Compute Engine virtual machine. The analysis was intended as an exploratory risk-stratification approach rather than as a validated individual prediction tool.

## 3. Results

### 3.1. Study Population Characteristics and Anti-Dengue IgG Seroprevalence

**Figure 1:**
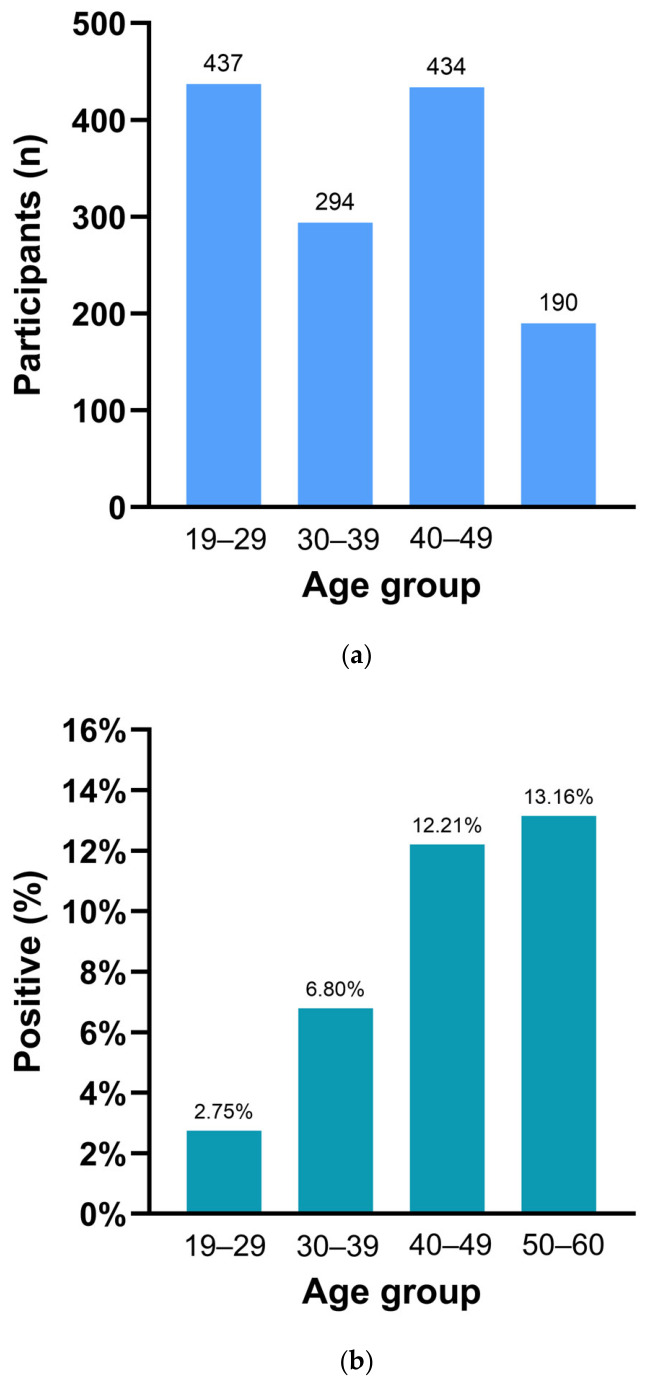
Age distribution and anti-dengue IgG seroprevalence by age group. (**a**) Number of participants in each age group, (**b**) Percentage of anti-dengue IgG-positive individuals in each age group. Seroprevalence increased progressively with age, from 2.75% in the 19–29-year group to 13.16% in the 50–60-year group.

Among the 1355 military personnel included in the analysis, participant age ranged from 19 to 60 years, with a median of 38 years (mean 36.8; SD 8.67) (Figure 1a).

Most participants belonged to the Navy (69.0%), followed by the Arma dei Carabinieri (29.8%), with the remainder drawn from other branches of the Armed Forces and a small group of civilian Defence staff. Participants were distributed across all age groups, with the 19–29 and 40–49-year groups accounting for the largest proportions of the cohort (32.25% and 32.03%, respectively; Figure 1a). Women accounted for 8.49% of the study population. Overall, 110 individuals tested positive for anti-dengue IgG antibodies, corresponding to a seroprevalence of 8.12%.

Based on self-reported travel history, 255 participants (18.82%) were classified as having been exposed to dengue-endemic regions, whereas 1100 (81.18%) were categorised as non-exposed (Table 1).

A contingency analysis compared the proportion of IgG-positive individuals between these two groups. Among exposed personnel, 63 of 255 (24.71%) tested positive for anti-dengue IgG. In contrast, only 47 of 1100 (4.27%) non-exposed individuals were seropositive. The association between prior exposure to endemic areas and IgG seropositivity was statistically significant (χ^2^ = 113.15, *p* < 0.0001).

Individuals with a history of exposure to dengue-endemic regions showed a substantially higher likelihood of IgG seropositivity, with an odds ratio of 7.35 (95% CI: 4.89–11.05), indicating markedly higher odds of anti-dengue IgG seropositivity among exposed personnel.

### 3.2. Age and Exposure Status in Relation to Anti-Dengue IgG Seropositivity

When participants were stratified into age groups (19–29, 30–39, 40–49 and 50–60 years), the distribution of seropositive individuals showed a clear increase with advancing age, with the highest proportion observed in the 50–60 age group (Figure 1b). Anti-dengue IgG seroprevalence rose progressively across age categories, from 2.75% in the youngest group to 13.16% in the oldest. A Cochran–Armitage trend test confirmed a statistically significant upward trend in seropositivity with increasing age (Z = 5.66, *p* < 0.0001).

**Figure 2:**
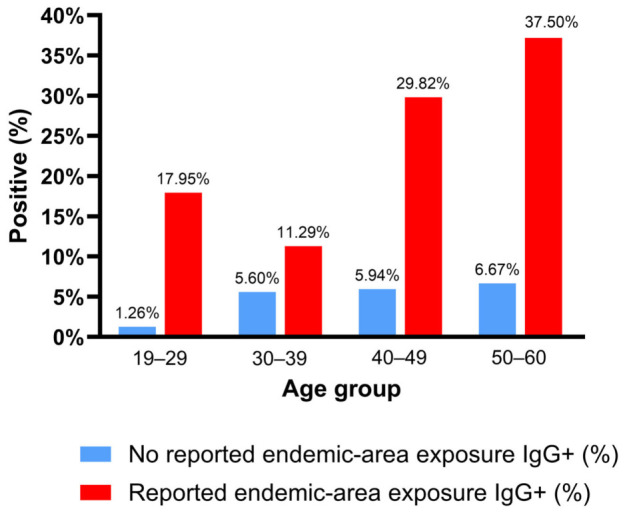
Anti-dengue IgG seropositivity by age group and reported exposure history. Bars show the percentage of anti-dengue IgG-positive individuals among participants with and without self-reported travel or deployment to dengue-endemic regions, stratified by age group.

Anti-dengue IgG seropositivity differed consistently between participants with and without reported travel or deployment to dengue-endemic regions within each age group (Figure 2). In the 50–60-year group, seropositivity was 37.50% among participants with reported endemic-area exposure and 6.67% among those with no reported endemic-area exposure. Similar differences were observed in the 40–49-year group (29.82% vs. 5.94%), the 30–39-year group (11.29% vs. 5.60%), and the 19–29-year group (17.95% vs. 1.26%).

The relationship between sex and anti-dengue IgG seropositivity was also examined. Women represented 8.49% of the study population. Seropositivity was detected in 8.20% of men and 7.27% of women. This difference was not statistically significant (χ^2^ = 0.066, *p* = 0.7976), indicating no statistically significant association between sex and IgG seropositivity. Finally, seroprevalence was compared between land-based forces (Army, Arma dei Carabinieri and Air Force) and sea-based forces (Navy). Anti-dengue IgG seropositivity was observed in 8.12% of land-based personnel and 8.24% of sea-based personnel, with no statistically significant difference between groups.

### 3.3. Cumulative Exposure Risk Score and Association with IgG Seropositivity

For each mission or deployment, a cumulative exposure risk score was assigned on the basis of the CDC classification of dengue transmission risk [26]. These scores were analysed in relation to anti-dengue IgG serostatus, as described in the Methods section and detailed in Supplementary S1_Data (sheets “Dataset_individual_level” and “Country_risk_classification”).

Preliminary analyses showed statistically significant associations between both exposure status and age with anti-dengue IgG seropositivity. To further explore these relationships, the distribution of dengue exposure risk levels was examined separately in IgG-positive and IgG-negative individuals. In addition to age-group comparisons, age and exposure-based risk level were therefore analysed as continuous variables to better characterise their relationship with IgG seropositivity.

**Figure 3:**
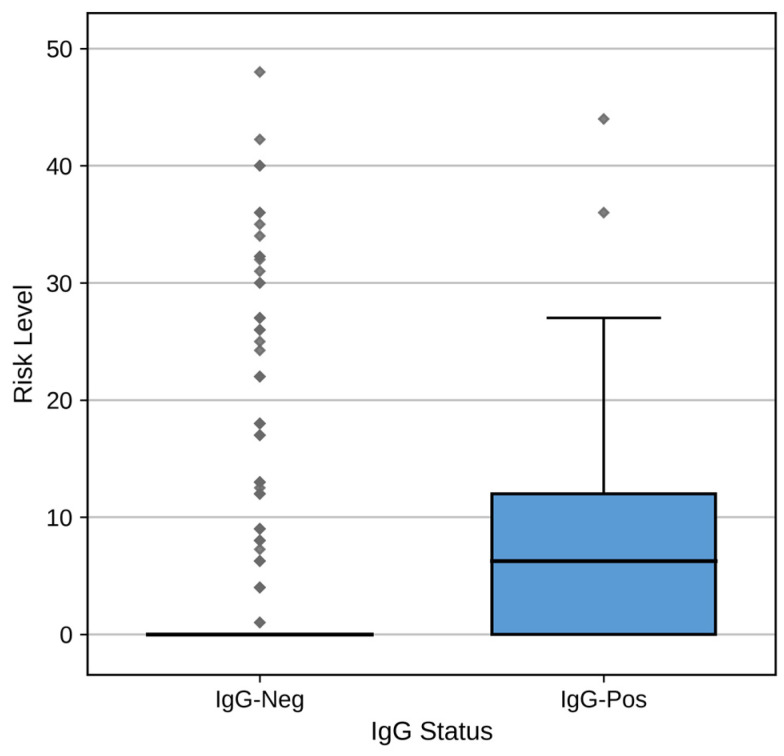
Cumulative exposure risk level in IgG-negative and IgG-positive individuals. Boxplots show the distribution of cumulative exposure risk scores according to anti-dengue IgG serostatus.

Boxplot analysis of cumulative risk scores revealed a marked difference between seronegative and seropositive participants (Figure 3). Among IgG-negative individuals, both the median risk score and the interquartile range (IQR) were 0.00, indicating that most observations clustered at the lowest exposure level. In contrast, IgG-positive individuals showed a broader distribution of risk values, with a median score of 6.25 and an IQR of 12.00.

The strength of the association between dengue seropositivity and cumulative risk score was assessed using Spearman’s rank correlation. A positive correlation was observed between IgG status (coded as 0 = negative and 1 = positive in S1_Data) and the exposure-based risk score (ρ = 0.299, *p* < 0.0001). Consistent results were obtained using the Mann–Whitney U test, which demonstrated a statistically significant difference in risk scores between IgG-positive and IgG-negative individuals (U = 38,965.0, *p* < 0.0001).

To contextualise the relative contribution of exposure and age, Spearman’s correlation was also calculated between IgG seropositivity and age. Although this association was statistically significant (ρ = 0.1517, *p* < 0.0001), it was weaker than the correlation observed for the exposure-based risk score (ρ = 0.299).

**Figure 4:**
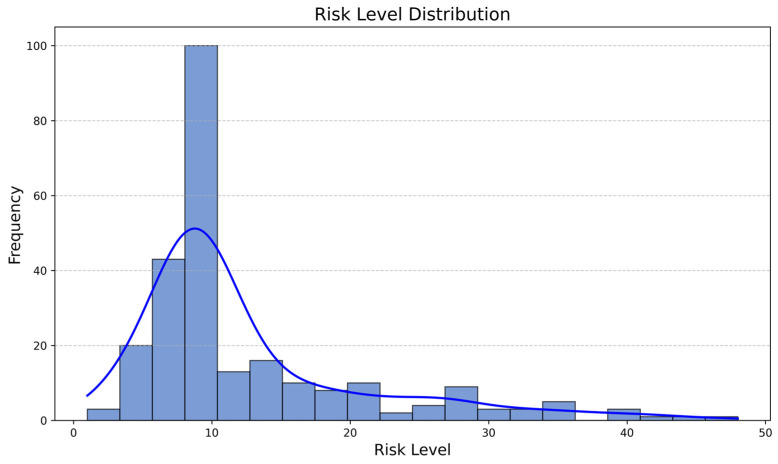
Distribution of non-zero cumulative dengue risk scores. Histogram showing the distribution of non-zero risk scores across the full study population. The blue curve represents a smoothed density curve of the distribution. The data display a right-skewed distribution, with a peak frequency around 6–10 and a long tail extending to higher values.

Finally, the distribution of non-zero risk scores was examined using a histogram restricted to individuals with measurable exposure (Figure 4). The resulting distribution was right-skewed, with most values concentrated between 6 and 10. Frequencies declined progressively at higher scores, although a small number of individuals reached values exceeding 30, indicating heterogeneous exposure patterns across the cohort.

### 3.4. Predictive Modelling

**Figure 5:**
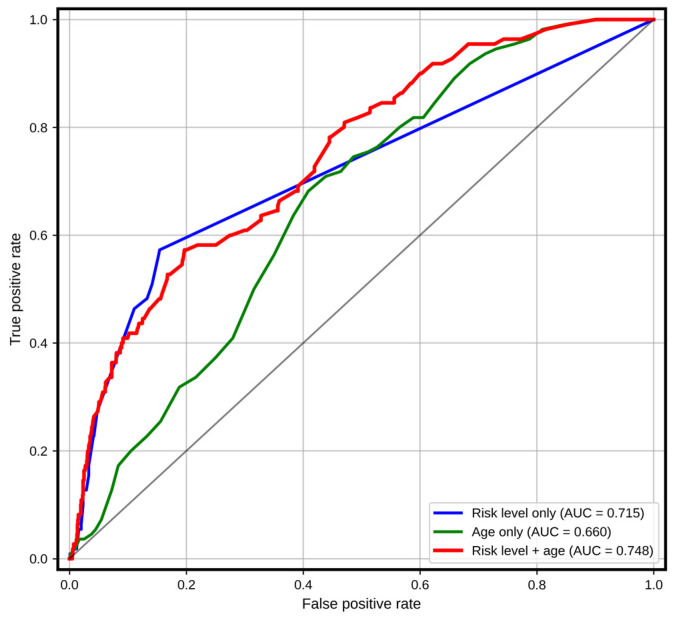
ROC curves for logistic regression models predicting IgG seropositivity. Receiver operating characteristic curves comparing the predictive performance of three logistic regression models for IgG seropositivity: exposure-based risk level only (AUC = 0.72), age only (AUC = 0.66), and the combined model including risk level and age (AUC = 0.75). The grey diagonal line represents random classification (AUC = 0.5). The combined model showed the highest AUC among the three logistic regression models.

To assess the independent and combined contributions of exposure-based risk level and age to anti-dengue IgG seropositivity, three logistic regression models were fitted: one including cumulative risk score only, one including age only, and a third incorporating both variables (Supplementary S1_Data, sheet “Dataset_individual_level”). The model based solely on cumulative risk score achieved an AUC of 0.72, indicating good discrimination between seropositive and seronegative individuals. The age-only model showed a lower AUC of 0.66, reflecting a weaker, albeit statistically significant, predictive contribution. When age and risk level were combined, model performance improved, yielding an AUC of 0.75 (Figure 5) with both predictors independently associated with IgG seropositivity (*p* < 0.05 for both variables). Although overall classification accuracy reached 91.51%, this metric is likely overestimated due to class imbalance, and the AUC was therefore considered a more appropriate measure of discriminative ability given the low seroprevalence observed in the study population. Logistic regression coefficients were 0.07495 for risk level and 0.03169 for age, with an intercept of −3.91.

**Figure 6:**
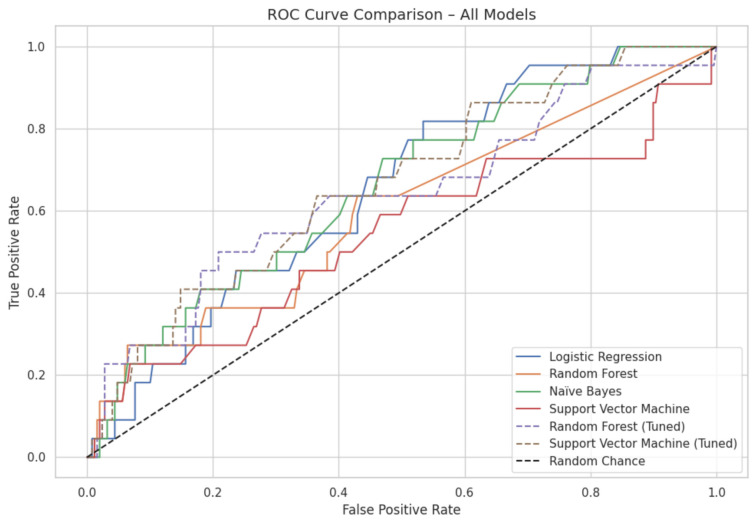
ROC curves for machine-learning models predicting IgG seropositivity. Receiver operating characteristic curves for baseline and tuned versions of logistic regression, random forest, naïve Bayes, and support vector machine (SVM) models trained on the same feature set (Age, Sex, Armed Forces branch, and Risk Level). Random forest showed the highest AUC post-hyperparameter tuning (AUC = 0.79), compared with logistic regression (AUC = 0.75), SVM (AUC = 0.72), and naïve Bayes (AUC = 0.68). The dashed diagonal line represents random classification.

Complementary analyses using supervised machine-learning classifiers trained on the same feature set supported these findings. Following hyperparameter optimisation, the random forest model achieved the highest discriminative performance on the stratified test set (AUC = 0.79; F1-score = 0.45), followed by logistic regression (AUC = 0.75), support vector machine (AUC = 0.72), and naïve Bayes (AUC = 0.68) (Figure 6). Full details on model specifications, parameter grids, and evaluation metrics are provided in Supplementary S2_File.

Exploratory PCA identified four components explaining the total variance. The first two components, primarily driven by age and exposure-based risk level, accounted for 66.7% of the variance. Additional details and visual outputs are provided in S2_File.

### 3.5. Geographical Exposure

**Figure 7:**
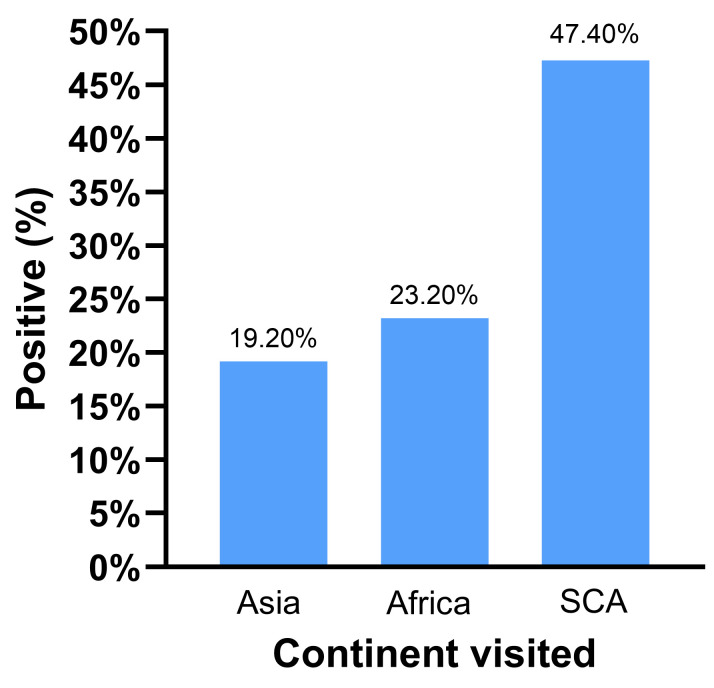
Anti-dengue IgG seroprevalence by continent of reported travel. Percentage of participants testing positive for anti-dengue IgG antibodies, among personnel who reported visiting dengue-endemic regions, categorised by continent of reported travel or deployment: Asia, Africa and South and Central America (SCA).

To provide a geographical perspective, the association between travel to dengue-endemic continents and anti-dengue IgG seropositivity was examined. Exposure was categorised by continent for all individuals reporting international travel, and statistical analyses were conducted to assess the relationship between geographical exposure and seropositivity. Travel to Asia, South and Central America (SCA), and Africa was significantly associated with increased odds of anti-dengue IgG seropositivity (Figure 7).

The highest seroprevalence was observed among subjects reporting travel to SCA, followed by Africa and Asia.

Specifically, the odds ratio was 10.794 for Asia (95% CI: 6.07–19.19, *p* < 0.0001), 10.399 for SCA (95% CI: 3.70–29.24, *p* < 0.0001), and 5.711 for Africa (95% CI: 3.81–8.56, *p* < 0.0001).

By contrast, travel to Europe was not significantly associated with anti-dengue IgG seropositivity, despite an elevated odds ratio, likely reflecting the limited number of exposed individuals (OR 5.645; 95% CI: 0.51–62.75, *p* = 0.5921). No association was observed for North America, where no seropositive cases were identified among travellers.

## 4. Discussion

This study provides a seroepidemiological assessment of anti-dengue IgG seropositivity among Italian military personnel deployed on international missions and identifies several relevant epidemiological and operational patterns. Overall, anti-dengue IgG seroprevalence in this cohort was 8.12%, indicating that a substantial proportion of personnel showed serological evidence compatible with previous exposure. Seroprevalence was markedly higher among individuals with a history of travel to dengue-endemic regions (24.71%) compared with those reporting no such exposure (4.27%), reinforcing the role of overseas deployment as a major determinant of anti-dengue IgG seropositivity, in line with previous evidence from deployed military populations [21,22,23].

Several unmeasured factors may further influence dengue exposure risk in deployed military personnel, including duration and season of deployment, type of field activity, intensity of vector exposure, and use of personal protective measures [27]. These variables were not available in the present dataset and may explain part of the residual variability in IgG seropositivity.

No association was observed between sex and anti-dengue IgG seropositivity, with comparable prevalence among male and female personnel, suggesting that sex did not influence IgG seropositivity in this cohort.

The highest proportion of seropositive individuals was observed in the 50–60-year age group. This likely reflects cumulative exposure linked to repeated international missions, particularly considering the involvement of Italian military personnel in overseas operations since the late 1980s, consistent with previous reports of dengue exposure among deployed military personnel [21,22,23]. Among participants aged 30 years or older, most had undertaken at least one foreign mission, which may partly explain the progressive increase in seroprevalence with age. Nevertheless, age alone provided only a partial explanation for seropositivity. Its predictive value was substantially enhanced when analysed in combination with the cumulative exposure risk score, suggesting that age may partly reflect cumulative travel- or deployment-related exposure rather than represent an independent determinant of seropositivity.

In the absence of detailed data on mission duration and time spent in endemic areas, it was not possible to directly quantify exposure intensity. However, Spearman’s rank correlation analysis demonstrated a moderate but robust association between anti-dengue IgG seropositivity and the CDC-derived cumulative dengue risk score, exceeding the correlation observed with age alone. This interpretation was supported by the Mann–Whitney U test, which showed significantly higher risk scores among IgG-positive compared with IgG-negative individuals. Multivariate logistic regression further quantified the relative contributions of exposure-based risk score alone (AUC = 0.72), age alone (AUC = 0.66), and their combination (AUC = 0.75), confirming improved discrimination when both variables were considered jointly. The positive regression coefficient associated with the risk score further supports the epidemiological usefulness of the CDC-derived cumulative dengue risk score in this cohort.

While an AUC of 0.75 reflects acceptable discriminative performance, it also indicates room for improvement. In this context, supervised machine-learning models provided a complementary assessment of the relationship between demographic and exposure-related variables and anti-dengue IgG seropositivity. Their performance was consistent with previous studies showing the potential value of machine-learning approaches in settings characterised by low disease prevalence or class imbalance [28,29]. Incorporation of the exposure-based score into machine-learning frameworks supported the value of individual-level risk stratification, although these models should be interpreted as exploratory tools rather than as validated standalone prediction systems.

In operational contexts where detailed epidemiological data may be limited, parsimonious models based on readily available variables can inform pre-deployment counselling and vaccination prioritisation for personnel assigned to higher-risk destinations [30]. These findings, reported to the military medical personnel of the Italian Armed Forces, supported the planning of targeted preventive measures, including structured pre-deployment counselling and prioritised vaccination strategies, particularly for individuals deploying to regions with higher dengue transmission risk such as Asia, South and Central America, and Africa.

From this perspective, measurement of anti-dengue IgG before deployment may represent a useful extension of the proposed approach, particularly for personnel assigned to dengue-endemic areas. Establishing a pre-mission serological baseline could help distinguish pre-existing seropositivity from possible post-deployment seroconversion and improve the interpretation of longitudinal surveillance data.

This model is most applicable to personnel from non-endemic or low-prevalence settings, where previous travel or deployment history has a greater role in explaining anti-dengue IgG seropositivity. In travellers from endemic areas, where background seroprevalence, cumulative lifetime exposure and age-seropositivity relationships may differ substantially, the model would require recalibration and external validation using local seroepidemiological data. A similar approach could also be adapted to other arthropod-borne infections, provided that reliable exposure categories and validated serological or molecular markers of infection are available [30].

Consistent with these analytical results, the markedly higher risk observed among personnel reporting travel or deployment to dengue-endemic regions provides strong evidence that overseas military missions significantly increase the likelihood of anti-dengue IgG seropositivity. Given the elevated seroprevalence observed in deployed personnel and the well-documented risk of ADE, which may exacerbate disease severity during subsequent infections, prioritisation of vaccination for military personnel operating in dengue-endemic regions appears justified. This recommendation is further supported by the availability of the live attenuated tetravalent vaccine TAK-003 (Qdenga^®^ Takeda, Osaka, Japan), licenced in Europe in December 2022 [31,32,33]. Long-term follow-up data (up to 4.5 years) demonstrated sustained efficacy of TAK-003 against virologically confirmed dengue (overall 61.2%) and against hospitalised virologically confirmed dengue (84.1%), with higher and more balanced protection across all four serotypes in baseline seropositive individuals. In baseline seronegative subjects, meaningful protection was observed against DENV-1 and DENV-2.

However, dengue vaccination in military personnel should be considered according to individual and mission-related risk, taking into account destination-specific risk, expected duration and recurrence of deployment, and baseline serostatus when available.

Geographical stratification of exposure revealed heterogeneity in dengue risk across deployment destinations, underscoring the importance of preventive strategies tailored to geographic risk profiles. Such targeted approaches may enhance protection of military personnel and help maintain operational readiness during international missions. The highest seroprevalence was observed among personnel reporting travel to South and Central America, where dengue may co-circulate with other arboviruses, including the flavivirus Zika virus and alphaviruses such as chikungunya and Mayaro viruses. This epidemiological context should be considered when interpreting serological results, particularly because co-circulating flaviviruses may contribute to serological cross-reactivity. In parallel, climate change and the expanding distribution of *A. albopictus* in Italy and other southern European countries increase the likelihood of dengue introduction and local transmission in previously non-endemic areas [34,35].

In this context, the finding that 4.27% of individuals without reported travel to endemic regions were IgG-positive warrants careful interpretation. Possible explanations include incomplete or inaccurate reporting of travel history, cross-reactivity of serological assays with antibodies elicited by other flaviviruses or prior yellow fever vaccination, and the potential underdiagnosis or underreporting of autochthonous dengue infections in Italy [10,11,12,13,14,15,16,17].

This point is particularly relevant because cross-reactivity is a well-described limitation of flavivirus serology and can complicate the interpretation of IgG-based assays in individuals with previous flavivirus infection or vaccination [36]. In personnel from European countries, potential serological confounders include West Nile virus, which has been documented in Italy [37], and Usutu virus, reported in Italy and other EU/EEA countries [38], previous tick-borne encephalitis virus infection or vaccination in selected individuals [39], and previous Japanese encephalitis vaccination as an additional flavivirus serological confounder [40]. Accordingly, ELISA seropositivity should be interpreted as serological evidence compatible with previous dengue exposure rather than as definitive proof of previous dengue infection.

As Italian military personnel deployed abroad may receive vaccine against yellow fever virus (YFV) for specific operational theatres, potential serological cross-reactivity in anti-dengue IgG ELISA results was considered. Previous studies [41] showed minimal cross-reactivity between YFV vaccine-induced antibodies and dengue IgG ELISA assays, suggesting that a substantial contribution to the observed seropositivity is unlikely. However, recent evidence indicates that the diagnostic specificity of dengue IgG ELISA after flavivirus vaccination may depend on the assay used, the vaccine considered and the time elapsed since vaccination [40].

Several limitations should be acknowledged. Clinical information during deployment and after return to Italy was not systematically collected for all participants. Therefore, IgG-positive participants cannot be classified as symptomatic or asymptomatic on the basis of the present data. However, no cases of symptomatic dengue confirmed by molecular testing were documented among participants included in the analysed cohort. The persistence of anti-dengue IgG antibodies supports their use as a marker of cumulative serological exposure, although it prevents attribution of seropositivity to a specific mission or travel episode. Confirmatory neutralisation assays, such as plaque reduction neutralisation testing (PRNT) or microneutralisation, were not performed. In future studies, neutralization testing would help clarify whether anti-dengue IgG seropositivity reflects previous dengue exposure in individuals with prior flavivirus infection or vaccination [36,39,40,41].

The cumulative exposure score, although necessarily simplifying a complex epidemiological reality, demonstrated a clear and statistically robust association with seropositivity, supporting its construct validity. The lack of detailed information on mission duration, timing, and seasonality likely attenuated the precision of exposure estimates. Integrating additional variables, including the nature of field activities, together with serological and environmental factors such as vector density, could further improve the predictive accuracy of the model and refine deployment-related dengue risk assessment in future studies. In addition, the focus on Italian Armed Forces personnel limits generalizability to civilian populations, given differences in demographics, travel patterns and exposure contexts. Underrepresentation of women and the age distribution of the cohort may also influence seroprevalence estimates. Finally, potential heterogeneity across military units or specific operational settings was not addressed.

The study also has several strengths. It includes a relatively large cohort of Italian military personnel evaluated within a defined pre-deployment setting, a population for which recent European seroepidemiological data remain limited [21,22,23]. The availability of individual-level travel and deployment information allowed exposure-based stratification, while the CDC-derived risk score provided a transparent approach to summarise cumulative geographical exposure. In addition, the integration of conventional epidemiological analyses with predictive modelling offers a reproducible framework that can be refined in future military and traveller-medicine studies.

Overall, the implementation of targeted preventive strategies for military personnel has the potential not only to protect individual health but also to reduce the importation of dengue cases and, consequently, the risk of autochthonous transmission in non-endemic countries where competent vectors are established.

## 5. Conclusions

This study provides a comprehensive seroepidemiological assessment of anti-dengue IgG seropositivity among Italian military personnel, showing that seropositivity was associated with previous travel or deployment to dengue-endemic regions. Cumulative exposure, as reflected by a CDC-derived risk score, was more strongly associated with seropositivity than age alone, while predictive modelling supported the value of exposure-based risk stratification and was consistent with the main epidemiological findings. These findings highlight the value of individual-level exposure-based risk stratification before deployment. Given the potential risk of severe disease during secondary dengue infection through mechanisms related to antibody-dependent enhancement, targeted preventive strategies, including risk-based vaccination, pre-deployment counselling, and enhanced surveillance, should be considered. Implementation of such measures may help protect the health of deployed personnel while reducing the risk of dengue importation and contributing to limiting autochthonous transmission in non-endemic European countries.

## Figures and Tables

**Table 1:** Anti-dengue IgG seropositivity according to prior exposure in Italian military personnel (n = 1355).

Exposure Status	Anti-Dengue IgG Positive, n	Anti-Dengue IgG Negative, n	Total, n
Exposed	63	192	255
Non-exposed	47	1053	1100
Total	110	1245	1355

## Data Availability

All relevant data supporting the findings of this study are within the manuscript and its Supporting Information files (S1_Data and S2_File).

## Supplementary Materials

The following supporting information can be downloaded at: https://www.mdpi.com/article/10.3390/tropicalmed11060167/s1, S1_Data contains anonymized dataset supporting all statistical analyses. This file includes anonymized individual-level information from all participants included in the study, comprising anti-dengue IgG serostatus, age, deployment-related exposure variables, and the CDC-derived cumulative dengue risk score. The dataset was used for statistical analyses, receiver operating characteristic curves, machine learning models, and figure generation. S2_File contains the Python scripts and corresponding outputs used for data preprocessing, exploratory data analysis, statistical analyses, machine learning model development and validation, and automated figure generation. All analyses were performed in Google Colab, a cloud-based interactive notebook environment, using Python and scikit-learn.

## Abbreviations

The following abbreviations are used in this manuscript:

DENV	Dengue virus
ADE	Antibody-dependent enhancement
GDPR	General Data Protection Regulation
NTU	NovaTec Units
ORs	Odds ratio
CIs	Confidence intervals
CDC	Centers for Disease Control and Prevention
SVM	Support vector machine
ROC	Receiver operating characteristic
AUC	Area under the curve
PCA	Principal component analysis
IDE	Integrated development environment
IQR	Interquartile range
SCA	South and Central America
PRNT	Plaque reduction neutralisation test
YFV	Yellow fever virus

## References

[1] Guzman M.G., Harris E. (2015). Dengue. Lancet.

[2] Merakou C., Amendola A., Fortuna C., Marsili G., Fiorentini C., Argentini C., Benedetti E., Rezza G., Maraglino F., Del Manso M. (2023). Diagnosis of Imported Dengue and Zika Virus Infections in Italy from November 2015 to November 2022: Laboratory Surveillance Data from a National Reference Laboratory. Viruses.

[3] Riccò M., Peruzzi S., Balzarini F., Zaniboni A., Ranzieri S. (2022). Dengue Fever in Italy: The “Eternal Return” of an Emerging Arboviral Disease. Trop. Med. Infect. Dis..

[4] Cochet A., Calba C., Jourdain F., Grard G., Durand G.A., Guinard A., Noël H., Paty M.C., Franke F., Auzet-Caillaud M. (2022). Autochthonous dengue in mainland France, 2022: Geographical extension and incidence increase. Euro Surveill..

[5] European Centre for Disease Prevention and Control Historical Data on Local Transmission of Dengue in the EU/EEA. https://www.ecdc.europa.eu/en/all-topics-z/dengue/surveillance-and-disease-data/autochthonous-transmission-dengue-virus-eueea-previous-years.

[6] Lazzarini L., Barzon L., Foglia F., Manfrin V., Pacenti M., Pavan G., Rassu M., Capelli G., Montarsi F., Martini S. (2020). First autochthonous dengue outbreak in Italy, August 2020. Euro Surveill..

[7] Barzon L., Gobbi F., Capelli G., Montarsi F., Martini S., Riccetti S., Sinigaglia A., Pacenti M., Pavan G., Rassu M. (2021). Autochthonous dengue outbreak in Italy 2020: Clinical, virological and entomological findings. J. Travel Med..

[8] Cassaniti I., Ferrari G., Senatore S., Rossetti E., Defilippo F., Maffeo M., Vezzosi L., Campanini G., Sarasini A., Paolucci S. (2023). Preliminary Results on an Autochthonous Dengue Outbreak in Lombardy Region, Italy, August 2023. Euro Surveill..

[9] European Centre for Disease Prevention and Control (ECDC) Communicable Disease Threats Report, Week 30, 2023. https://www.ecdc.europa.eu/sites/default/files/documents/communicable-disease-threats-report-week-30-2023_0.pdf.

[10] Istituto Superiore di Sanità (ISS) Casi di Dengue in Italia: I Dati Aggiornati. Arbovirosi in Italia 2023. https://www.epicentro.iss.it/arbovirosi/2023.

[11] De Carli G., Carletti F., Spaziante M., Gruber C.E.M., Rueca M., Spezia P.G., Vantaggio V., Barca A., De Liberato C., Romiti F. (2023). Outbreaks of Autochthonous Dengue in Lazio Region, Italy, August to September 2023: Preliminary Investigation. Euro Surveill..

[12] Vita S., Bordi L., Sberna G., Caputi P., Lapa D., Corpolongo A., Mija C., D’Abramo A., Maggi F., Vairo F. (2024). Autochthonous Dengue Fever in 2 Patients, Rome, Italy. Emerg. Infect. Dis..

[13] Salvo P.F., Baldin G., Raffaelli F., Ciccullo A., Borghetti A., Tamburrini E., Ricci R., Di Donato M., Di Giambenedetto S., Torti C. (2024). Autochthonous Dengue Outbreak in Rome, Italy, in 2023. J. Travel Med..

[14] Istituto Superiore di Sanità (ISS) Dengue Dashboard. EpiCentro—Arbovirosi in Italia. https://www.epicentro.iss.it/arbovirosi/dashboard-2023.

[15] Istituto Superiore di Sanità (ISS) Dengue Dashboard. EpiCentro—Arbovirosi in Italia. https://www.epicentro.iss.it/arbovirosi/dashboard-2024.

[16] Sacco C., Liverani A., Venturi G., Gavaudan S., Riccardo F., Salvoni G., Fortuna C., Marinelli K., Marsili G., Pesaresi A. (2024). Autochthonous Dengue Outbreak in Marche Region, Central Italy, August to October 2024. Euro Surveill..

[17] European Centre for Disease Prevention and Control (ECDC) Seasonal Surveillance of Dengue in the EU/EEA-Weekly Report, Week 47, 2025. https://www.ecdc.europa.eu/en/dengue/surveillance-and-updates/seasonal-surveillance-dengue-eueea-weekly.

[18] Roy S.K., Bhattacharjee S. (2021). Dengue Virus: Epidemiology, Biology, and Disease Aetiology. Can. J. Microbiol..

[19] Rothman A.L. (2011). Immunity to Dengue Virus: A Tale of Original Antigenic Sin and Tropical Cytokine Storms. Nat. Rev. Immunol..

[20] Guzman M.G., Alvarez M., Halstead S.B. (2013). Secondary Infection as a Risk Factor for Dengue Hemorrhagic Fever/Dengue Shock Syndrome: An Historical Perspective and Role of Antibody-Dependent Enhancement of Infection. Arch. Virol..

[21] Peragallo M.S., Nicoletti L., Lista F., D’Amelio R. (2003). Probable Dengue Virus Infection among Italian Troops, East Timor, 1999–2000. Emerg. Infect. Dis..

[22] Hesse E.M., Martinez L.J., Jarman R.G., Lyons A.G., Eckels K.H., De La Barrera R.A., Thomas S.J. (2017). Dengue Virus Exposures among Deployed U.S. Military Personnel. Am. J. Trop. Med. Hyg..

[23] Agboli E., Jöst H., Frangoulidis D., Song L.H., Anh D.D., Katsounas A., Velavan T.P., Schmidt-Chanasit J. (2026). Dengue in Deployed Military Personnel, 1905–2024: A Systematic Review of Incidence, Diagnostics and Prevention. J. Travel Med..

[24] Yavari Nejad F., Varathan K.D. (2021). Identification of Significant Climatic Risk Factors and Machine Learning Models in Dengue Outbreak Prediction. BMC Med. Inform. Decis. Mak..

[25] Mazhar B., Ali N.M., Manzoor F., Khan M.K., Nasir M., Ramzan M. (2024). Development of Data-Driven Machine Learning Models and Their Potential Role in Predicting Dengue Outbreak. J. Vector Borne Dis..

[26] Centers for Disease Control and Prevention Areas with Risk of Dengue. https://www.cdc.gov/dengue/areas-with-risk/index.html.

[27] Duvignaud A., Stoney R.J., Angelo K.M., Chen L.H., Cattaneo P., Motta L., Gobbi F.G., Bottieau E., Bourque D.L., Popescu C.P. (2024). Epidemiology of Travel-Associated Dengue from 2007 to 2022: A GeoSentinel Analysis. J. Travel Med..

[28] Zhao N., Charland K., Carabali M., Nsoesie E.O., Maheu-Giroux M., Rees E., Yuan M., Garcia Balaguera C., Jaramillo Ramirez G., Zinszer K. (2020). Machine Learning and Dengue Forecasting: Comparing Random Forests and Artificial Neural Networks for Predicting Dengue Burden at National and Sub-National Scales in Colombia. PLoS Negl. Trop. Dis..

[29] da Silva Neto S.R., Tabosa Oliveira T., Teixeira I.V., Aguiar de Oliveira S.B., Souza Sampaio V., Lynn T., Endo P.T. (2022). Machine Learning and Deep Learning Techniques to Support Clinical Diagnosis of Arboviral Diseases: A Systematic Review. PLoS Negl. Trop. Dis..

[30] Leder K., Steffen R., Cramer J.P., Greenaway C. (2015). Risk Assessment in Travel Medicine: How to Obtain, Interpret, and Use Risk Data for Informing Pre-Travel Advice. J. Travel Med..

[31] European Medicines Agency Dengue Tetravalent Vaccine (Live, Attenuated) Takeda—Opinion on Medicine for Use Outside EU. https://www.ema.europa.eu/en/opinion-medicine-use-outside-EU/human/dengue-tetravalent-vaccine-live-attenuated-takeda.

[32] Tricou V., Yu D., Reynales H., Biswal S., Saez-Llorens X., Sirivichayakul C., Lopez P., Borja-Tabora C., Bravo L., Kosalaraksa P. (2024). Long-Term Efficacy and Safety of a Tetravalent Dengue Vaccine (TAK-003): 4.5-Year Results from a Phase 3, Randomised, Double-Blind, Placebo-Controlled Trial. Lancet Glob. Health.

[33] World Health Organization (2024). WHO Position Paper on Dengue Vaccines. Wkly. Epidemiol. Rec..

[34] Rezza G. (2012). Aedes albopictus and the Reemergence of Dengue. BMC Public Health.

[35] Frasca F., Sorrentino L., Fracella M., D’Auria A., Coratti E., Maddaloni L., Bugani G., Gentile M., Pierangeli A., d’Ettorre G. (2024). An Update on the Entomology, Virology, Pathogenesis, and Epidemiology Status of West Nile and Dengue Viruses in Europe (2018–2023). Trop. Med. Infect. Dis..

[36] Chan K.R., Ismail A.A., Thergarajan G., Raju C.S., Yam H.C., Rishya M., Sekaran S.D. (2022). Serological cross-reactivity among common flaviviruses. Front. Cell. Infect. Microbiol..

[37] Rizzo C., Napoli C., Venturi G., Pupella S., Lombardini L., Calistri P., Monaco F., Cagarelli R., Angelini P., Bellini R. (2016). West Nile Virus Transmission: Results from the Integrated Surveillance System in Italy, 2008 to 2015. Euro Surveill..

[38] Angeloni G., Bertola M., Lazzaro E., Morini M., Masi G., Sinigaglia A., Trevisan M., Gossner C.M., Haussig J.M., Bakonyi T. (2023). Epidemiology, Surveillance and Diagnosis of Usutu Virus Infection in the EU/EEA, 2012 to 2021. Euro Surveill..

[39] Vilibic-Cavlek T., Ferenc T., Vujica Ferenc M., Bogdanic M., Potocnik-Hunjadi T., Sabadi D., Savic V., Barbic L., Stevanovic V., Monaco F. (2022). Cross-Reactive Antibodies in Tick-Borne Encephalitis: Case Report and Literature Review. Antibodies.

[40] Schnabel I., Schneitler S., Schüttoff T., Trawinski H., Lübbert C., Jassoy C. (2023). Diagnostic specificity of two dengue virus IgG ELISAs after yellow fever and Japanese encephalitis virus vaccination. Trop. Med. Infect. Dis..

[41] Souza N.C.S.E., Félix A.C., de Paula A.V., Levi J.E., Pannuti C.S., Romano C.M. (2019). Evaluation of serological cross-reactivity between yellow fever and other flaviviruses. Int. J. Infect. Dis..

